# Why Are Old-Age Disabilities Decreasing in Sweden and Denmark? Evidence on the Contribution of Cognition, Education, and Sensory Functions

**DOI:** 10.1093/geronb/gbac118

**Published:** 2022-09-16

**Authors:** Andreea C Badache, Elina Mäki-Torkko, Stephen Widen, Stefan Fors

**Affiliations:** School of Health Sciences, Faculty of Medicine and Health, Örebro University, Örebro, Sweden; Swedish Institute for Disability Research, Örebro University, Örebro, Sweden; School of Medical Sciences, Örebro University, Sweden; Audiological Research Center, Faculty of Medicine and Health, Örebro University, Örebro, Sweden; School of Health Sciences, Faculty of Medicine and Health, Örebro University, Örebro, Sweden; Aging Research Center, Karolinska Institutet and Stockholm University, Stockholm, Sweden; Centre for Epidemiology and Community Medicine, Stockholm, Sweden

**Keywords:** ADL limitations, IADL limitations, Nordic countries, Older adults, Sensory function

## Abstract

**Objectives:**

Improvements in educational attainment, cognitive and sensory functions, and a decline in the prevalence of disabilities have been observed in older adults in Sweden and Denmark. In the present study, it was investigated whether better cognition, higher educational attainment, and improved sensory function among older adults aged 60 and older in these countries have contributed to decreasing rates of old-age disabilities.

**Methods:**

The analyses were based on repeated cross-sectional data from the Survey of Health, Ageing, and Retirement in Europe for the 2004–2017 period. Descriptive data were used to benchmark the declining prevalence of disabilities, improving cognitive and sensory functions, and increased educational level. The association between time and disabilities was analyzed with logistic regression models, and the contribution of the improved cognitive function, education, and sensory function to the declining prevalence of old-age disabilities was estimated using the Karlson–Holm–Breen method for mediation analysis.

**Results:**

The analysis suggests that the declining prevalence of old-age disabilities in Sweden and Denmark between 2004 and 2017 can largely be attributed to improved cognitive function and vision and to a lesser extent by education and hearing ability.

**Discussion:**

These findings raise important questions about the causal mechanisms producing the associations between cognition, education, and sensory functions and disability in older age. Future studies should explore the causal nature of the associations between these mediators and old-age disabilities. In addition, they should explore whether these findings differ across regional and cultural contexts and over different time periods.

Globally, declining fertility rates and significant gains in life expectancy have led to an aging population. In the world, between 2015 and 2030, the number of older people aged 60 years and older is projected to grow by 56%, and by 2050, the global population of older persons is projected to have doubled its size, reaching nearly 2.1 billion (United Nations Department of Economic and Social Affairs, 2016). The population aged 80 and older is growing at a faster rate than younger age groups and is expected to triple by 2050 (United Nations Department of Economic and Social Affairs, 2016).

Disabilities occur in the intersection between the individual’s functional capacity and the environmental demands (Verbrugge & Jette, 1994). The disablement process in old age tends to differ from the processes leading to disabilities earlier in life. Evidence from life-course epidemiology suggests that differences in the risk of old-age disabilities may stem from exposures and experiences throughout the life course (Liu et al., 2010).

Due to living longer lives, people can expect to get more age-related diseases and disabilities; nevertheless, disability prevalence varies across countries (Beller & Epping, 2021; Lee et al., 2019). Perhaps the most robust evidence of decreasing prevalence of old-age disabilities comes from studies of the oldest old in the Nordic countries, where a series of studies has shown that the prevalence of limitations in activities of daily living (ADL) as well as in instrumental activities of daily living (IADL) has decreased over time (Ahrenfeldt et al., 2018; Christensen et al., 2013; Hossin et al., 2017). Another recent study found a decreasing trend in Hungary, Spain, and Poland; increasing trends in Belgium, France, and Germany; and a stagnation in Finland, Great Britain, and Sweden (Beller & Epping, 2021). These findings are paradoxical because during the same periods, the prevalence of morbidities in Sweden has increased (Hossin et al., 2017), and objectively measured physical function has remained stable or improved slightly (Christensen et al., 2013; Hossin et al., 2017). In Southern Europe, no clear trends have been observed for the rates of ADL/IADL limitations (Ahrenfeldt et al., 2018).

Cognitive functioning is one of the strongest predictors of functional impairment in old age (Clemmensen et al., 2020). Although the prevalence of disabilities is decreasing throughout the Nordic countries, average cognition seems to be improving in the population (Trahan et al., 2014), and these trends seem to persevere into old age (Christensen et al., 2013; Rönnlund & Nilsson, 2008) across Europe (Ahrenfeldt et al., 2018). This trend is in line with the “Flynn effect,” which refers to a continuous increase in intelligence across subsequent birth cohorts observed throughout the 20th century. These improvements correspond to about 3–5 IQ points per decade (Teasdale & Owen, 2005; Trahan et al., 2014). Recent findings suggest that the Flynn effect has weakened and potentially reversed in later-born cohorts, possibly due to environmental factors (Bratsberg & Rogeberg, 2018). However, for cohorts born between 1909 and 1969 in Sweden (Rönnlund & Nilsson, 2008) and up to 1979 in Denmark, the Flynn effect can still be observed (Teasdale & Owen, 2008).

Conversely, sensory impairments (hearing and vision) are common causes of ADL disabilities in older ages (Raina et al., 2004). In Sweden, during the 1992–2009 period, the rate of cataract surgeries rose from 4.47 to 9.00 per 1,000 inhabitants (Behndig et al., 2011). A similar trend was observed in Denmark (Kessel et al., 2011). Similarly, hearing acuity has improved significantly over four decades in Sweden (Hoff et al., 2018).

During the 20th century, most high-income countries went through a large educational expansion, leading to higher educational attainment (Breen, 2010). People with more education tend to live longer and healthier lives than those without (Case & Deaton, 2021; Tsai, 2017). Educational attainment is associated with labor market success, living conditions, access to health care, income, lifestyle choices, cognitive and noncognitive capabilities, health, and disability throughout the course of life (Tsai, 2017). Education has also been found to be associated with a reduction in the risk of cognitive impairment (Beller et al., 2022; Clouston et al., 2020) and education expansion is probably partly responsible for the Flynn effect (Bratsberg & Rogeberg, 2018). However, it has been suggested that the improvements in cognitive functioning due to education cannot fully explain the Flynn effect (Bratsberg & Rogeberg, 2018).

Greater educational attainment has also been associated with a decreased risk of ADL and IADL disabilities among older adults (Schoeni et al., 2008) and sensory functions decline (Chou et al., 2015). Furthermore, sensory impairments are risk factors for age-related cognitive decline, and potentially sensory–cognitive association can be explained by social isolation caused by sensory loss which in turn is a risk factor for cognitive decline (Hämäläinen et al., 2019). Thus, there are most likely causal feedback loops between education, cognition, and sensory function operating throughout the life course that may serve to amplify or attenuate the risk of old-age disabilities.

The Nordic countries are known for their resemblances shaped by a common history which has led to shared traditions and norms. Moreover, Denmark and Sweden have similar climates and political systems, described by welfare states and universal health care systems and comparable disability trends have been observed in both countries (Magnussen, 2010). It has been suggested that the observed improvements in ADL function in later-born cohorts of older adults observed in the Nordic countries might be driven by improved cognitive function (Christensen et al., 2013; Hossin et al., 2017). Other factors that might have contributed to the decline in old-age disabilities could be the educational expansion, improved treatments (e.g., cataract surgery; Behndig et al., 2011; Maharani et al., 2018), improved standards of living, lifestyle factors, and better rehabilitation measures (Christensen et al., 2013). A Danish study has also indicated that later-born cohorts had both better cognitive function and fewer disabilities, despite no reductions in morbidities and no improvement in measured physical function (Christensen et al., 2013). Hossin et al. (2017) explored this further and found evidence of an attenuation of the morbidity–disability link among older adults in Sweden between 1992 and 2011 (Hossin et al., 2017).

We hypothesize that better cognition, higher educational attainment, and improved sensory function among older adults in Sweden and Denmark have contributed to decreasing rates of old-age disabilities (measured by ADL and IADL). To achieve enough statistical power to address the research question and due to Survey of Health, Ageing and Retirement in Europe (SHARE) data availability, we pool data from Sweden and Denmark as there is robust evidence that the prevalence of old-age disabilities has decreased over time in both countries.

In the present study, we will test these hypotheses using data from national surveys of older adults in Sweden and Denmark, gathered during the period 2004–2017.

## Method

### Data

The study is based on data from SHARE (Börsch-Supan et al., 2013). SHARE is a cross-national and longitudinal study, designed to investigate the life-course effects of health, social, economic, and environmental policies of 140,000 European citizens aged 50+ (SHARE User Conference 2022, n.d.). Most interviews took place in the participants’ home; however, when a respondent was not able to do an interview due to health reasons, proxy interviews were conducted with relatives or caregivers. However, some parts of the questionnaire, including the cognitive tests, were not completed during proxy interviews. The SHARE samples were drawn at household level and the response rate differed for Sweden (53.7%) and Denmark (63.2%) in 2004–2005 (Bergmann et al., 2019) with a slight decrease in response rates across time in both countries (Bergmann et al., 2019). Refresher samples were constantly added during all follow-up waves except 2017, to compensate for attrition, to keep the sample representative, and to increase the sample size. In this study, we included all participants aged 60 and older, both those who participated in the following waves: 2004/2005, 2006/2007, 2011, 2013, 2015, and 2017, as well as those added through the refreshment samples, as the study uses a repeated cross-sectional design. The 2009 sample (Wave 3) consisted of SHARELIFE retrospective life questionnaires and did not contain measures of cognition and ADL/IADL limitations; therefore, it was not included in this study. In 2017 (wave 7) in addition to the regular panel questionnaire also a SHARELIFE questionnaire for all respondents who did not participate in Wave 3 was conducted; however, we included only those who participated in the regular panel.

After removing participants with missing data on any of the variables, the analytical sample consists of 23,315 observations, from 9,219 unique individuals, collected in 2004/2005, 2006/2007, 2011, 2013, 2015, and 2017. A flow chart of the participants selection can be found in Supplementary Figure 1.

### Procedures and Measures

#### Time

Time was measured as a categorical variable, denoting each wave by the year the study was conducted.

#### Disability

In this study, we define disability as difficulty in performing activities of daily living, measured through the self-reported scores on ADL adapted from Katz et al. (1970) and IADL adapted from Lawton and Brody (1969). The ADL index consists of six items: dressing, eating, cutting up food, using the toilet, walking across a room, and bathing/showering, and the IADL limitations were assessed by seven activities: using a map, preparing a hot meal, shopping for groceries, making telephone calls, taking medications, doing work around the house/garden, and managing finances. For both the ADL and IADL indexes, the participants who reported inability to perform one or more tasks, without help, were classified as having ADL and/or IADL limitations. Both ADL and IADL were classified into two categories, either no limitations or limitations in one or more tasks.

#### Cognitive function

Cognitive function was evaluated by three commonly used cognitive tests. *Immediate recall* measures how many of 10 words the respondent could name immediately after the interviewer reads the words. *Delayed recall* measures the ability to recall the same words after answering some other interview questions. *Verbal fluency* measures the number of animals that the respondent could name in one minute. We used the scores from the cognitive tests to compute a cognitive composite score (CCS). The composite score was calculated by standardizing each single test score before including them in a principal component analysis to generate a single score. The CCS was standardized, with a mean of 10 and an *SD* of 1.5 to mimic a reduced version of an ordinary IQ test, with a mean of 100 and an *SD* of 15. If there was missing information for an individual in any one of the cognitive tests, the score was coded as missing and excluded from the analysis. To assess the internal consistency of the three items comprising the CCS, a Cronbach alpha based on the standardized items was calculated and yielded an estimate of α = .78.

#### Sensory function

The sensory function comprises self-rated hearing ability and vision (eyesight) ability. Vision ability was measured by asking the participants “Is your eyesight [using glasses or contact lenses as usual]?” and hearing “Is your hearing [using a hearing aid as usual]?” Then they were asked to rate their ability on a 5-point Likert scale as 1 = excellent, 2 = very good, 3 = good, 4 = fair, 5 = poor.

#### Education

Educational level was measured through self-reported highest educational achievement which was categorized into three levels: primary and lower secondary (ISCED 0–2), upper secondary (ISCED 3–4), and tertiary (ISCED 5–6) following the International Standard Classification of Education 1997 (UNESCO, 1998). The six ISCED educational levels consist of ISCED 0 pre-primary level of education, ISCED 1 primary level of education, ISCED 2 lower secondary level of education, ISCED 3 upper secondary level of education, ISCED 4 postsecondary, nontertiary education, ISCED 5 first stage of tertiary education, and ISCED 6 second stage of tertiary education.

#### Covariates

In this study, age, sex, and country were included in the models as covariates.

### Statistical Analysis

The analyses were conducted in three steps. As a first step, descriptive data to benchmark the important empirical regularities that underpin our hypotheses were tabulated. That is, the declining prevalence of old-age disabilities, improving cognitive function, increased educational level, and improving sensory function. In the next step, the association between time and disabilities with adjustments for sex, age, and country were analyzed with logistic regression analyses. The results from these analyses are presented in terms of predicted probabilities extracted from logistic regressions through marginal standardization. This requires that the predicted probabilities sum to a weighted average reflecting the covariates’ distribution in the target population (Muller & MacLehose, 2014). By computing predicted probabilities, the results can be made more tangible. The results of the predicted probabilities are based on the reduced model (adjustments for sex, age, and country) and the full model (sex, age, country, cognitive function, education, and sensory functions) covariates.

Finally, the contribution of improved cognitive function, education, and sensory function to the declining prevalence of old-age disabilities was estimated. This was done using the Karlson–Holm–Breen (KHB) method for mediation analysis (Karlson et al., 2012). Due to the noncollapsibility of odds ratios, it is not possible to compare the estimates of odds ratios from nested models calculated separately (Mood, 2009). The KHB method solves this problem and yields robust estimates that are comparable across nested models. These models also provide an estimate of how much of the association between the main exposure and the outcome can be attributed to the mediators (Karlson et al., 2012). Robust standard errors that allow for intragroup correlation were calculated to account for nonindependent observations across the study waves (i.e., that some individuals participated in several waves). Moreover, the individual weights developed by the SHARE team were included in all analyses.

To estimate the contribution of the cognitive and sensory functions, the total “effect” of time on disabilities in the population was decomposed into direct and indirect “effects.” The “indirect effect” can be interpreted as the part of the initial association between time and disabilities that can be attributed to cognitive function, education, and sensory function, whereas the “direct effect” can be interpreted as the residual association remaining when accounting for cognition, education, and sensory function. This type of decomposition of regression coefficients is not straightforward in nonlinear regression models due to a rescaling problem caused by not separately identifying the coefficients and the error variance in nonlinear regression models (Karlson & Holm, 2011). However, this issue is resolved by the KHB method that allows for comparisons of estimates from nested logistic models (Karlson et al., 2012). With this method, the trends estimated from the comprehensive model (full or adjusted) are compared and decomposed to assess and quantify the contribution of each “mediator” (cognition, education, and sensory functions) to the overall trend (Karlson et al., 2012). Therefore, in this study, we decompose the trend of ADL and IADL limitations into a part that can be attributed to cognition, education, and sensory functions (the “indirect effect”) and a residual part (the “direct effect”; Karlson et al., 2012). The KHB output shows the estimated effect of the reduced model (total effect), the full model (direct effect), and the estimated differences between these two (indirect effect). Besides the estimates for the reduced and the full model, the KHB method also calculates the confounding ratio and the confounding percentage (Karlson et al., 2010), which gives a percentage measure for the proportion of the association that can be attributed to each of the “mediators.” A confounding percentage exceeding 100% indicates that without the observed changes in the distribution of the “mediators” the development would have gone in the other direction. In our case, that would mean that without increasing education and improved cognitive and sensory functions, the prevalence of disabilities would have increased rather than decreased. Yet, such an interpretation should be validated by checking the confidence intervals for the “direct effects.”

Although mediation analysis is commonly used to analyze the impact of intermediate variables on a causal effect of an exposure on an outcome, it is used and interpreted in a different manner in this study. Rather than decomposing a supposedly causal effect of an exposure on an outcome, into direct and indirect effects, we are estimating how much of an observed population-level change could be attributed to compositional changes in the population, in terms of the distribution of cognitive abilities, educational attainment, and sensory function. This application of mediation analysis has important implications for the interpretation of the estimates. The estimated “direct effect” is not to be considered a causal effect of time on the risk of old-age disabilities, but rather as residual, unexplained variance. Similarly, the “indirect” effect should be interpreted as the part of the observed association between time and risk of old-age disabilities that could be attributed to compositional changes in the distribution of the “mediators” in the population over time.

For all analyses, we used Stata (v.17) and the user-written *khb* command for the mediation analysis (Kohler et al., 2011). To reduce the impact of nonresponse and sample attrition on estimates, we use the cross-sectional calibrated weights developed by SHARE in all analyses.

## Results

### Study Population

There were slightly more participants from Sweden than from Denmark (56.8% vs. 43.3%) and more female than male participants (53.3% vs. 46.7%). Table 1 shows the weighted prevalence of ADL and IADL disability, the mean and range of the CCS and sensory function tests, age, educational attainment, stratified by wave, country, and sex and for the total population.

**Table 1.** Demographic Characteristics (Weighted Sample) of People Participating in 2004–2005, 2006–2007, 2011, 2013, 2015, 2017 SHARE study (Percentages, Weighted Numbers, and Range)

	Denmark							Sweden							Both Countries						
	2004/ 2005	2006/ 2007	2011	2013	2015	2017	Total	2004/ 2005	2006/ 2007	2011	2013	2015	2017	Total	2004– 2005	2006– 2007	2011	2013	2015	2017	Total
*N*	*942*	*1,579*	*1,353*	*2,573*	*2,424*	*1,177*	*10,084*	*1,847*	*1,928*	*1,615*	*3,558*	*3,263*	*1,011*	*13,231*	*2,789*	*3,507*	*2,968*	*6,131*	*5,687*	*2,188*	*23,315*
*%*							*43*							*57*							*100*
Sex																					
Male	415	710	617	1,206	1,131	539	4,603	826	898	762	1,699	1,525	443	6,143	1,241	1,609	1,381	2,907	2,657	979	10,754
Female	527	869	736	1,367	1,302	665	5,481	1,021	1,030	853	1,859	1,740	575	7,088	1,548	1,898	1,587	3,224	3,041	1,243	12,561
%	*55.9*	*55.1*	*54.4*	*53.1*	*53.5*	*55.3*	*54.4*	*55.3*	*53.4*	*52.8*	*52.3*	*53.3*	*56.5*	*53.6*	*55.5*	*54.1*	*53.5*	*52.6*	*53.4*	*55.9*	*53.9*
Age																					
Mean	*71.2*	*71.3*	*71.5*	*71.5*	*71.7*	*72.9*	*71.6*	*72*	*71.6*	*70.6*	*71.2*	*72*	*74.6*	71.8	71.7	71.5	*70.9*	71.3	71.9	73.8	71.7
Range	*60–97*	*60–99*	*60–100*	*60–101*	*60–101*	*60–101*	*60–101*	*60–101*	*60–97*	*60–99*	*60–100*	*60–102*	*60–105*	*60–105*	*60–101*	*60–99*	*60–100*	*60–101*	*60–102*	*60–105*	*60–105*
Education																					
Primary and lower secondary (%)	33.4	28.9	22.3	22.1	19.6	16.3	23.7	63.8	58.6	47.9	42.7	39.4	42	48.3	53	46.1	37.8	35	32.2	30	38.7
Upper secondary (%)	42.1	40.9	41.7	40.6	40.7	40.7	41.1	20.3	23.2	27.8	30.2	31.5	29.5	27.5	28.1	30.7	33.2	34.1	34.9	34.7	32.8
Tertiary (%)	24.4	30.2	36	37.2	39.8	43.1	35.30	17.22	18.2	24.5	27.1	29.1	28.5	24..2	18.9	23.2	29	30.9	33	35.3	28.5
ADL limitations																					
No limitations (%)	*87*	*89*	*90.5*	*89.2*	*90.3*	*90*	*89.4*	*88.4*	*89.4*	*87.8*	*91.1*	*90.6*	*89.6*	*89.7*	*87.9*	*89.2*	*88.8*	*90.4*	*90.5*	*89.8*	*89.6*
1+ limitations (%)	*12.9*	*11*	*9.6*	*10.7*	*9.7*	*10*	*10.6*	*11.7*	*10.6*	*12.2*	*8.9*	*9.5*	*10.5*	*10.3*	*12.1*	*10.8*	*11.2*	*9.6*	*9.6*	*10.2*	*10.4*
IADL limitations																					
No limitations (%)	*78.3*	*82.5*	*84.1*	*83.1*	*82.5*	*80.6*	*82.1*	*79.7*	*84.9*	*85.2*	*87*	*84.8*	*84.4*	*84.6*	*79.2*	*83.9*	*84.8*	*85.5*	*83.9*	*82.7*	*83.6*
1+ limitations (%)	*21.7*	*17.5*	*159*	*16.9*	*17.6*	*19.4*	*7.9*	*20.3*	*15.1*	*14.8*	*13*	*15.3*	*15.6*	*15.4*	*20.8*	*16.1*	*15.7*	*14.5*	*16.1*	*17.3*	*16.4*
Cognitive function																					
Mean	*9.6*	*9.8*	*10.1*	*10.1*	*10.2*	*10.2*	*10*	*9.5*	*9.8*	*10*	*9.9*	*10.1*	*10*	*9.9*	*9.5*	*9.8*	*10*	*9,9*	*10,1*	*10,1*	*9,9*
Range	*5.2–14.1*	*5.2–15*	*5.2–14.8*	*5.2–14.4*	*5.2–14.7*	*5.2–15.3*	*5,2–15.3*	*5.2–14.2*	*5.2–14.1*	*5.2–14.8*	*5.2–15.1*	*5.2–14.6*	*5.2–14.1*	*5.2–15.1*	*5.2–14.2*	*5.2–15*	*5.2–14.8*	*5.2–15.1*	*5.2–14.7*	*5.2–15.3*	*5.2–15.3*
Sensory functions																					
** **Hearing																					
** **Mean	2.6	2.5	2.4	2.5	2.4	2.4	2.5	2.6	2.5	2.5	2.6	2.6	2.6	2.6	2.6	2.5	2.5	2.5	2.5	2.5	2.5
** **Range	*1–5*							*1–5*							*1––5*						
** **Vision																					
** **Mean	2.4	2	2.2	*2*	*2*	2	2.1	2.6	2.4	2.3	2.3	2.3	2.3	2.3	2.6	2.2	2.2	2.2	2.2	2.2	2.2
** **Range	*1–5*							*1–5*							*1–5*						

*Notes:* ADL = activities of daily living; IADL = instrumental activities of daily living.

### Predicted Probabilities

**Figure 1.**
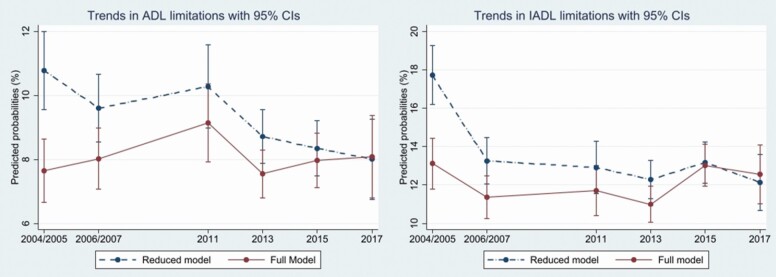
(Right) The predicted probability of ADL for Sweden and Denmark, between 2004 and 2017; (left) trends in IADL for Sweden and Denmark, between 2004 and 2017, for older people aged 60 and older, with 95% confidence intervals, adjusted for sex, age, and country. The reduced model is adjusted for sex, age, and country, and the full model is adjusted for sex, age, country, cognitive function, education, and sensory functions. ADL = activities of daily living; IADL = instrumental activities of daily living.

The estimated predicted probabilities of the regression analysis for ADL and IADL disabilities across time and by country are presented in Figure 1 with adjustments for covariates (sex, age, and country) in the reduced model and with further accounting for the mediators (cognitive function, education, and sensory functions) in the full model. For both ADL and IADL limitations, overall, there was a decrease in the prevalence between 2004 and 2017 (see Figure 1).

The predicted probability of having ADL limitations has been decreasing over the period, from 12% in 2004–2005 to 9.1% in 2017. For IADL limitations, the corresponding drop went from 20.7% in 2004–2005 to 14.9% in 2017.

**Figure 2.**
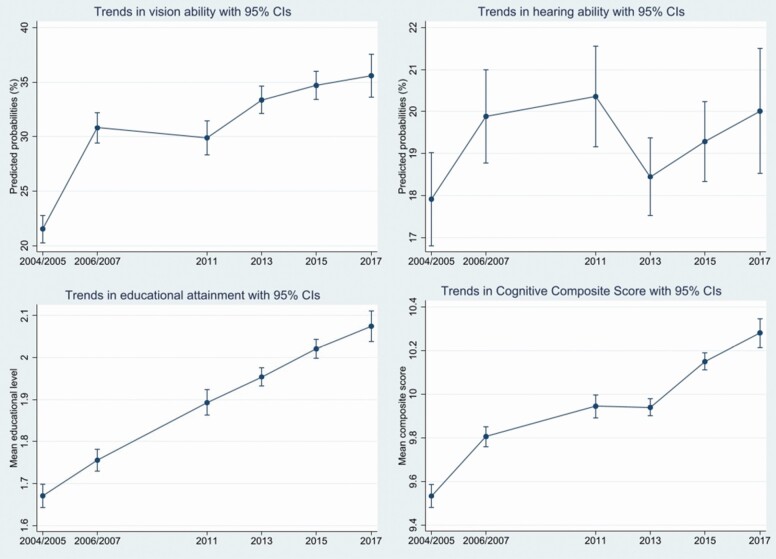
Trends in hearing ability (upper right) and vision ability (upper left) for Sweden and Denmark combined between 2004 and 2017; trends in the cognitive composite score (CCS) between 2004 and 2017 in Denmark and Sweden (lower right) combined, trends in the educational attainment between 2004 and 2017 for Sweden and Denmark combined (lower left) for older people aged 60 and older, with 95% confidence intervals, adjusted for sex, age, and country.

Meanwhile, the cognitive scores increased between 2004 and 2017. Over the same period, the predicted probabilities of reporting good vision abilities (21.5%–35.6%) have been increasing in both countries and the predicted probability of reporting good hearing ability (17.9%–20%) has also been increasing between 2004/2005 and 2017. Moreover, between 2004 and 2017, there are also a growing number of older people with higher levels of education (see Figure 2). The regression estimates can be found in Supplementary Table 3.

### Decomposition Analysis

The results from the decomposition analyses are reported in Table 2 for ADL limitations and in Table 3 for IADL limitations and by age groups in Supplementary Table 1 and gender in Supplementary Table 2. The odds ratios (OR) and 95% confidence intervals (CI), together with the estimates from the reduced models (total effect), show the difference in the odds of reporting ADL/IADL between 2004 and the subsequent survey years when adjusting for age, sex, and country. The estimates from the full model (direct effect) show the association between the period and ADL/IADL limitations when further accounting for cognition, education, and sensory functions. When cognition, education, and sensory functions are added to the full models, the relative change between the reduced and the full model is quantified in the “confounding percentage.” The confounding percentage gives the percentage of the original association that can be attributed to education, cognitive function, and sensory function.

**Table 2.** Decomposition Analysis Results of the Total Time Effect on ADL Limitations Into Direct and Indirect Effects Through Education, Cognitive Function, and Sensory Function Using Karlson–Holm–Breen Method for Logistic Regression

	Both countries		Denmark		Sweden	
	OR	95% CI	OR	95% CI	OR	95% CI
2004–2005	(Base outcome)		(Base outcome)		(Base outcome)	
** **Total effect (reduced model)	1	[1.1]	1	[1.1]	1	[1.1]
** **Direct effect (full model)	1	[1.1]	1	[1.1]	1	[1.1]
** **Indirect effect (difference)	1	[0.93, 1.07]	1	[0.89, 1.12]	1	[0.92, 1.09]
2006–2007						
** **Total effect (reduced model)	0.88	[0.75, 1.03]	0.81	[0.64, 1.03]	0.91	[0.74, 1.13]
** **Direct effect (full model)	1.07	[0.91, 1.25]	1.03	[0.81, 1.31]	1.08	[0.87, 1.34]
** **Indirect effect (difference)	0.83***	[0.77, 0.89]	0.79***	[0.70, 0.889]	0.85***	[0.77, 0.93]
Confounding ratio and percentage	−2.1 (148.6%)	—	−7.1 (114%)	—	−1.1 (187.7%)	—
		% Mediated	% Mediated		% Mediated	
Cognitive function	49.3	—	46.9	—	51.6	—
Education	5.2	—	2.6	—	6.7	
Vision	38.7	—	42.8	—	35.4	—
Hearing	6.7	—	7.7	—	6.3	—
2011						
** **Total effect (reduced model)	0.95	[0.79, 1.14]	0.65**	[0.49, 0.845]	1.18	[0.94, 1.50]
** **Direct effect (full model)	1.23*	[1.02, 1.48]	0.89	[0.67, 1.17]	1.47^**^	[1.16, 1.87]
** **Indirect effect (difference)	0.78***	[0.72, 0.84]	0.73***	[0.65, 0.82]	0.80***	[0.73, 0.88]
Confounding ratio and percentage	−0.2 (528.5%)		3.6 (72.2%)		−0.4 (131.4%)	
	% Mediated		% Mediated		% Mediated	
Cognitive function	56.5	—	66.1	—	48.4	—
Education	10.4	—	4.3	—	15.9	
Vision	27.2	—	21.5	—	32.0	—
Hearing	5.9	—	8.1	—	3.7	—
2013						
** **Total effect (reduced model)	0.78**	[0.67, 0.92]	0.78*	[0.61, 0.99]	0.77*	[0.63, 0.95]
** **Direct effect (full model)	1.02	[0.87, 1.20]	1.13	[0.88, 1.45]	0.96	[0.77, 1.19]
** **Indirect effect (difference)	0.77^***^	[0.71, 0.83]	0.69***	[0.61, 0.78]	0.81***	[0.73, 0.89]
Confounding ratio and percentage	−11.2 (108.9%)		−2.1 (147.2%)		6.3 (84.3%)	
	% Mediated		% Mediated		% Mediated	
Cognitive function	53.2		59.7		46.3	
Education	12.6		3.9		23.1	
Vision	33.1		30.8		34.9	
Hearing	1.1		6.1		−4.4	
2015						
** **Total effect (reduced model)	0.75^***^	[0.64, 0.88]	0.67^**^	[0.52, 0.86]	0.79*	[0.64, 0.98]
** **Direct effect (full model)	1.08	[0.91, 1.28]	1.04	[0.80, 1.35]	1.10	[0.88, 1.37]
** **Indirect effect (difference)	0.70^***^	[0.64, 0.76]	0.65^***^	[0.57, 0.74]	0.72^***^	[0.65, 0.80]
Confounding ratio and percentage	−3.9 (125.3%)	—	−10.6 (109.4%)	—	−2.6 (138.3%)	
	% Mediated		% Mediated		% Mediated	
Cognitive function	59.1	—	62.3	—	55.4	—
Education	11.4		4.1		18.9	
Vision	27.3	—	27.5	—	27.2	—
Hearing	2.1	—	6.1	—	−1.6	—
**2017**						
** **Total effect (reduced model)	0.72^**^	[0.58, 0.88]	0.65^**^	[0.48, 0.87]	0.75*	[0.56, 0.99]
** **Direct effect (full model)	1.10	[0.89, 1.36]	1.06	[0.78, 1.44]	1.11	[0.82, 1.50]
** **Indirect effect (difference)	0.65^***^	[0.60, 0.71]	0.61^***^	[0.54, 0.70]	0.67^***^	[0.60, 0.75]
** **Confounding ratio and percentage	−3.7 (127.3%)		−7.3 (113.7%)		−2.8 (135.7%)	
	% Mediated		% Mediated		% Mediated	
Cognitive function	60.8	—	64.3	—	57.8	—
Education	11.2		4.8		16.7	
Vision	25.1	—	25	—	25.7	—
Hearing	2.9	—	5.9	—	−0.2	—
*N*	23,315		10,084		13,231	

*Notes:* ADL = activities of daily living; CI = confidence interval; OR = odds ratio. The reduced models were adjusted for age, sex, and country; the full models were additionally adjusted also for the mediators. Confounding ratio gives information on the total effect size relative to the direct effect size, calculated by total effect/direct effect. Confounding percentage measures the percentage change of effect attributable to confounding net of rescaling, calculated by indirect effect/total effect. CI between brackets.

*p < .05,

^**^ *p* < .01,

^***^ *p* < .001.

**Table 3.** Decomposition Analysis Results of the Total Time Effect on IADL Limitations Into Direct and Indirect Effects Through Education, Cognitive Function, and Sensory Function—using Karlson–Holm–Breen Method for Logistic Regression

	Both countries		Denmark		Sweden	
	OR	95% CI	OR	95% CI	OR	95% CI
2004–2005	(Base outcome)		(Base outcome)		(Base outcome)	
** **Total effect (reduced model)	1	[1, 1]	1	[1, 1]	1	[1, 1]
** **Direct effect (full model)	1	[1, 1]	1	[1, 1]	1	[1, 1]
** **Indirect effect (difference)	1	[0.93, 1.08]	1	[0.89, 1.12]	1	[0.91, 1.10]
2006–2007						
** **Total effect (reduced model)	0.70^***^	[0.61, 0.80]	0.73^**^	[0.60, 0.88]	0.69***	[0.57, 0.83]
** **Direct effect (full model)	0.86*	[0.75, 0.99]	0.94	[0.78, 1.14]	0.82*	[0.68, 0.10]
** **Indirect effect (difference)	0.81^***^	[0.76, 0.88]	0.77***	[0.68, 0.87]	0.83^***^	[0.76, 0.92]
Confounding ratio and percentage	2.3 (57.7%)	—	8.1 (80.2%)	—	1.9 (48,1%)	—
	% Mediated		% Mediated		% Mediated	
Cognitive function	47.3	—	41.2	—	52.6	—
Education	2,9		2.1		3.2	—
Vision	45.3	—	50.5	—	40.4	—
Hearing	4.6	—	6.2	—	3.9	—
2011						
** **Total effect (reduced model)	0.68^***^	[0.58, 0.80]	0.60^***^	[0.48, 0.75]	0.74^**^	[0.60, 0.92]
** **Direct effect (full model)	0.88	[0.75, 1.04]	0.83	[0.66, 1.03]	0.94	[0.75, 1.16]
** **Indirect effect (difference)	0.77^***^	[0.71, 0.83]	0.73^***^	[0.65, 0.82]	0.80^***^	[0.72, 0.88]
Confounding ratio and percentage	3.1 (67.8%)	—	2.7 (62.6%)	—	4.3 (77.1%)	—
	% Mediated		% Mediated		% Mediated	
Cognitive function	56.6	—	62.2	—	51.7	—
Education	5.9		3.8		7.8	
Vision	33.3	—	27,1	—	38.1	—
Hearing	4.2	—	6.9	—	2.4	—
2013						
** **Total effect	0.64^***^	[0.56, 0.73]	0.70^**^	[0.57, 0.86]	0.60^***^	[0.50, 0.72]
** **Direct effect	0.84*	[0.73, 0.97]	1.03	[0.83, 1.27]	0.75^**^	[0.62, 0.90]
** **Indirect effect	0.76^***^	[0.70, 0.82]	0.68^***^	[0.60, 0.77]	0.80^***^	[0.72, 0.88]
Confounding ratio and percentage	2.6 (61.9%)	—	−14.8 (106.8%)	—	1.8 (43.7%)	—
	% Mediated		% Mediated		% Mediated	
Cognitive function	52.4	—	54.7	—	49.6	—
Education	7.1	—	3.3	—	11.4	—
Vision	39.7	—	37.6	—	38.1	—
Hearing	0.8	—	4.7	—	2.4	—
2015						
** **Total effect (reduced model)	0.70^***^	[0.61, 0.80]	0.71^**^	[0.57, 0.88]	0.69^***^	[0.57, 0.83]
** **Direct effect (full model)	1.01	[0.88, 1.17]	1.11	[0.90, 1.38]	0.96	[0.80, 1.16]
** **Indirect effect (difference)	0.69^***^	[0.63, 0.75]	0.64^***^	[0.56, 0.73]	0.71^***^	[0.64, 0.79]
Confounding ratio and percentage	−26 (103.9%)	—	−3.2 (131%)	—	10.2 (90.2%)	—
	% Mediated		% Mediated		% Mediated	
Cognitive function	58.9	—	57.4	—	59.1	—
Education	6.5		3.5		9.3	—
Vision	33.2	—	34	—	32.6	—
Hearing	1.5	—	5.1	—	−1	—
2017						
** **Total effect (reduced model)	0.63^***^	[0.53, 0.74]	0.74*	[0.58, 1.94]	0.54^***^	[0.42, 0.69]
** **Direct effect (full model)	0.97	[0.82, 1.16]	1.22	[0.95, 1.56]	0.82	[0.63, 1.05]
** **Indirect effect (difference)	0.64^***^	[0.59, 0.70]	0.61^***^	[0.53, 0.69]	0.66^***^	[0.59, 0.74]
Confounding ratio and percentage	16.9 (94.1%)	—	−1.5 (165.6%)	—	3 (66.9)	—
	% Mediated		% Mediated		% Mediated	
Cognitive function	60.9	—	59.8	—	61.4	—
Education	6.4	—	4.2	—	8.2	—
Vision	30.7	—	31.1	—	30.5	—
Hearing	2	—	5	—	−0.1	—
*N*	23,315		10,084		13,231	

*Notes:* CI = confidence interval; IADL = instrumental activities of daily living; OR = odds ratio. The reduced models were adjusted for age, sex, and country; the full models were additionally adjusted also for the mediators. Confounding ratio gives information on the total effect size relative to the direct effect size, calculated by total effect/direct effect. Confounding percentage measures the percentage change of effect attributable to confounding net of rescaling, calculated by indirect effect/total effect. CI between brackets.

*p < .05,

^**^ *p* < .01,

^***^ *p* <.001.

The results suggest statistically significant decreases in the odds of reporting ADL limitations between 2004 and 2017 for both countries combined (OR = 0.72, *p* < .01) and in Denmark (OR = 0.65, *p* < .01) and Sweden (OR = 0.75, *p* < .05). A statistically significant difference (indirect effect) between the reduced and full models was observed for ADL limitations (OR = 0.65, *p* < .001), which can be attributed to the mediators. By disentangling the contribution of each mediator, most of the improvements in ADL limitations observed between 2004 and 2017 could be attributed to improved cognitive function (60.8%), vision (25.1%), education (11.2%), and hearing (2.9%). However, in Denmark, cognitive function (64.3%) plays a bigger role than in Sweden (57.8%), whereas the role played by education is more important in Sweden (16.7%) compared with in Denmark (4.8%). Thus, the declining rates of ADL limitations over time can be entirely attributed to cognition, education, and sensory functions as indicated by the confounding percentage (127.3%; see Table 2). Similarly, when grouped by gender, the odds of reporting ADL limitations has decreased for both females and males (OR = 0.79, *p* > .05), but statistically significant only in females (OR = 0.67, *p* < .01); however, there are statistically significant differences (indirect effect) which can be attributed to the mediators in both groups (males: OR = 0.67, *p* < .001; females: OR = 0.64, *p* < 0001).

Likewise, the odds of reporting IADL limitations decreased between 2004 and 2017 in both countries (OR = 0.63, *p* < .001) and the difference between the full and reduced models is also statistically significant in IADL (OR = 0.64, *p* < .001). Similarly, in both countries, most of the decline in IADL disabilities can be attributed to cognition (60.9%), vision (30.7%), education (6.4%), and hearing (2.0%). The confounding percentage shows that, in total, 94% of the decline in IADL limitations over the period can be attributed to improvements in cognition, education, and sensory function (see Table 3). Similarly, when grouped by gender, the odds of reporting IADL limitations has decreased for both genders (males: OR = 0.67, *p* < .01; females: OR = 0.60, *p* < .001), and there also statistically significant differences in the indirect effects (males: OR = 0.61, *p* < .001; females: OR = 0.66, *p* < .001), which can be attributed to the mediators.

Over the 2004–2017 period, most of the difference between the full and reduced models (indirect effect) can be explained by cognitive function and less by the other mediators. The role of cognition is even more accentuated in the 75+ age groups for both ADL (74.5%) and IADL (65.3%) limitations, followed by vision, education, and hearing (see Supplementary Table 1). Moreover, in the 60–74 older adults’ group, for the ADL limitations, education (30.6%) plays a more important role than vision (26.5%).

## Discussion

The aim of this study was to investigate whether the observed declining prevalence of ADL and IADL limitations observed in Sweden and Denmark can be explained by improvements in education, cognitive function, and sensory functions among older adults (60+) during the 2004–2017 period. The results suggest that the declining prevalence of ADL and IADL can be almost entirely attributed to these factors, with improved cognitive function and vision ability being the most important drivers, followed by education and hearing, for both genders. As evidenced by the results, the role of cognition is increasingly more important in older ages, among both males and females.

Some important limitations and strengths of this study should be kept in mind when interpreting the results. Most measures used were self-reported, which could yield biased results if the subjective propensity to report disabilities have changed over time (Podsakoff et al., 2003). The relatively low response rates and moderate levels of attrition may potentially generate sample selection biases, limiting the representativeness of the database and the generalizability of the results; however, the overall response rate of SHARE is quite high when compared with U.S. or other European surveys (Börsch-Supan et al., 2013). The provision of calibrated weights helps to minimize the potential selection bias generated by unit nonresponse and panel attrition. On the other hand, the study is based on random samples of the older population in Sweden and Denmark, which makes it possible to track national disability trends over time; it includes community-dwelling and older people living in nursing homes and includes harmonized outcomes across the different national settings. Furthermore, a limitation of the used KHB model is that it does not account for potential interactions between the exposure (time) and the mediators (cognition function, education, sensory function).

Another crucial caveat is that these are not causal analyses. Now, we do not assume that the association between time and disabilities is causal. There is likely nothing in time itself that has led to decreasing rates of old-age disabilities, but these improvements are more likely caused by changes that have occurred during the time passed. Thus, the association between time and the mediators should not be interpreted in causal terms. Instead, we suggest that improved cognitive function, higher education, and better sensory function may be the very changes that have caused this decline in old-age disabilities. The results support this hypothesis. Although we cannot rule out that the association between the mediators (cognition function, education, sensory function) and the outcome (old-age disabilities) is noncausal and driven by unmeasured confounders, we argue—based on the previous literature—that there is good reason to believe that cognition and sensory function are causally associated with disabilities in old age. The evidence for a causal association between education and old-age disabilities is more ambiguous.

Previous studies have shown that the prevalence of ADL and IADL disabilities is decreasing over time in the Nordic countries (Ahrenfeldt et al., 2018; Christensen et al., 2013; Hossin et al., 2017) and in many other parts of the world (Fuller-Thomson et al., 2009); however, large differences in cognitive functions and disabilities have been observed across European regions (Formanek et al., 2019). In old age, the Nordic countries have been reported as having the highest cognitive performance of all Europeans, whereas southern Europeans show the lowest cognitive performance (Ahrenfeldt et al., 2018; Formanek et al., 2019; Skirbekk et al., 2012). Improved cognition has been suggested as a cause for improved ADL and IADL limitations (Ahrenfeldt et al., 2018; Christensen et al., 2013), a hypothesis that was tested and supported by the findings of this study.

Our results showed that more improvements in old-age disabilities could be attributed to education in the 60–74 age group than in the 75+ group. This may suggest that education is becoming increasingly associated with disabilities in later-born cohorts of older adults. This is in line with a recent Swedish study, showing that the educational inequalities in disability-free life expectancy in old age increased in Sweden between 2002 and 2014 (Sundberg et al., 2022).

As sensory losses are risk factors for physical and functional disability in older people, we also hypothesized that improvements in sensory functions in older adults in the Nordic countries may have contributed to the decreasing rates of disabilities. The results partly support this hypothesis and show that vision played a bigger role than both hearing ability and education. A potential explanation for this finding could be that vision loss from cataract has been dramatically reduced due to increased access to cataract surgery for older adults over the past decades. Both in Sweden and Denmark there has been a dramatic rise in the rate of cataract surgeries (Behndig et al., 2011). Furthermore, the survey questions on sensory functions include the use of assistive devices (glasses and hearing aids) and despite increased age-related vision and hearing loss, it is also becoming increasingly more common for older people to use aids (e.g., hearing aids and glasses; McCormack & Fortnum, 2013); thus, they would be more likely to self-report better hearing and vision, which seems to be in line with our findings, which showed improvements over time on sensory functions. However, when it comes to hearing, despite being fitted for a hearing aid, many still do not use their aids; therefore, the smaller improvements in hearing could be partially explained by this (McCormack & Fortnum, 2013).

The current study tests an important hypothesis of whether the declining rates of ADL and IADL disabilities observed in Sweden and Denmark can be explained by improved education, cognitive function, and sensory function in later-born cohorts. Likewise, the findings of the current study of decreased prevalence of old-age disabilities in Sweden and Denmark are in line with the results from previous studies of trends in the prevalence of old-age disabilities in the Nordic countries (Ahrenfeldt et al., 2018; Christensen et al., 2013; Hossin et al., 2017). As early life exposures and environmental influences are likely important and affect aging processes, we can expect the cognitive function of older adults to keep increasing for quite some time, yet studies from Sweden and Denmark have been reporting increases in the IQ scores for cohorts born in the 1970s (Rönnlund et al., 2013) and 1980s (Teasdale & Owen, 2000) even though they are smaller than for previous birth cohorts. When health care and nutrition improve, increases in intellectual abilities tend to be observed in the population (Daley et al., 2003). However, a causal effect of nutrition and health on intelligence is still under debate as diet and nutrition are not unique in enhancing or maintaining cognitive ability (Flynn, 2009). Furthermore, this trend could be also explained by the “cognitive reserve” hypothesis, which describes why those with higher education, IQ, occupational attainment or highly engaged in leisure activities experience fewer cognitive changes in the presence of age-related brain pathology and helps individuals to cope by recruiting compensatory processes that slow down cognitive decline in aging (Stern, 2009). The findings from this study suggest that this may yield a substantial dividend in terms of old-age disabilities. Future studies should explore the causal nature of the associations between cognition function, education, sensory function, and old-age disabilities. In addition, they should seek to explore whether these findings replicate across different regional and cultural contexts and over different time periods. Moreover, further research could investigate whether there is a “net effect” of education and cognition on the risk of old-age disabilities that is not mediated via physical function and to what extend the positive role of education, cognition, and sensory functions in old-age disabilities is mediated by the use of medical, technical, and assistive devices.

## Supplementary Material

gbac118_suppl_Supplementary_Material
