# Supplementary material for: Why Are Old-Age Disabilities Decreasing in Sweden and Denmark? Evidence on the Contribution of Cognition, Education, and Sensory Functions
Source: J Gerontol B Psychol Sci Soc Sci. 2022 Sep 16;78(3):483–95. doi: 10.1093/geronb/gbac118 (PMC9985323; doi:10.1093/geronb/gbac118)
Supplement: gbac118_suppl_Supplementary_Material [file gbac118_suppl_supplementary_material.pdf]

SUPPLEMENTATY MATERIAL

Table 1. Decomposition analysis results of the total time effect on ADL and IADL limitations into direct and indirect effects through education, cognitive and sensory functions– using Karlson-Holm-Breen (KHB) method for logistic regressions, stratified by age groups

	ADL limitations				IADL limitations			
	Sweden and Denmark				Sweden and Denmark			
	Age band 75+		Age band 60-74		Age band 75+		Age band 60-74	
	OR	95% CI	OR	95% CI	OR	95% CI	OR	95% CI
**2004-2005**								
Total effect	Base outcome		Base outcome		Base outcome		Base outcome	
Direct effect	Base outcome		Base outcome		Base outcome		Base outcome	
Indirect effect	Base outcome		Base outcome		Base outcome		Base outcome	
**2006-2007**								
Total effect	0.79*	[0.63,0.99]	0.98	[0.78,1.22]	0.72**	[0.59,0.88]	0.65***	[0.54,0.79]

Direct effect	0.95	[0.76,1.20]	1.19	[0.91,1.53]	0.90	[0.74,1.11]	0.80*	[0.65,0.97]
Indirect effect	0.83**	[0.72,0.94]	0.82***	[0.79,0.90]	0.80**	[0.70,0.92]	0.812***	[0.74,0.90]
**Confounding ratio and percentage**	5 (79.9%)	-	-0.1 (796%)	-	3.2 (68.4%)	-	1.9 (46.8%)	-
	**% Mediated**		**% Mediated**		**% Mediated**		**% Mediated**	
Cognitive function	58.2	-	40,	-	49.2	-	43.8	-
Education	-0.4		13	-	0.2		7.5	
Vision	36	-	40.8	-	48.3	-	41.7	-
Hearing	6.2	-	6.2	-	2.3	-	7.1	-
**2011**								
Total effect	0.74*	[0.57,0.96]	1.18	[0.91,1.53]	0.69**	[0.55,0.86]	0.64***	[0.51,0.81]
Direct effect	0.95	[0.73,1.22]	1.56**	[1.158,2.03]	0.91	[0.73,1.14]	0.83	[0.66,1.05]

Indirect effect	0.78***	[0.68,0.90]	0.76***	[0.69,0.84]	0.76***	[0.66,0.87]	0.77***	[0.70,0.85]
**Confounding ratio and percentage**	5.5 (82%)	-	0.4 (-167.1%)	-	4 (75.1%)	-	2.4 (58.8%)	
	**% Mediated**		**% Mediated**		**% Mediated**		**% Mediated**	
Cognitive function	64.5	-	45.1	-	57.1	-	51.8	
Education	-0.9		23.9	-	0.4		14.5	
Vision	27.9	-	26.6	-	39.2	-	28.5	
Hearing	8.5	-	4.4	-	3.3	-	5.2	
**2013**								
Total effect	0.63***	[0.49,0.79]	0.99	[0.79,1.25]	0.56***	[0.46,0.68]	0.73**	[0.60,0.89]
Direct effect	0.80	[0.63,1.02]	1.34*	[1.05,1.70]	0.75**	[0.61,0.91]	0.96	[0.79,1.17]
Indirect effect	0.78***	[0.68,0.89]	0.74***	[0.67,0.82]	0.75***	[0.65,0.86]	0.76***	[0.69,0.84]

**Confounding ratio and percentage**	2.1 (53.1%)	-	-0.03 (-3500.8%)	-	2 (49.9%)	-	8.2 (87.9%)	-
	**% Mediated**		**% Mediated**		**% Mediated**		**% Mediated**	
Cognitive function	71.5	-	37.2	-	60.1	-	44.0	-
Education	-1.1		28.5		0.5		17.8	
Vision	29.6	-	33.2	-	39.4	-	36.7	-
Hearing	-0.02	-	1.9	-	-0,01	-	1.3	-
**2015**								
Total effect	0.63***	[0.50,0.79]	0.92	[0.72,1.17]	0.66***	[0.54,0.80]	0.75***	[0.61,0.91]
Direct effect	0.88	[0.69,1.11]	1.36	[1.05,1.80]	0.97	[0.79,1.18]	1.088	[0.88,1.34]
Indirect effect	0.72***	[0.62,0.82]	0.67***	[0.60,0.75]	0.68***	[0.59,0.78]	0.69***	[0.62,0.77]
**Confounding ratio and percentage**	3.5(71.7%)	-	-0.3 (458.4%)	-	12.2 (91,8%)	-	-3.4 (129.1%)	-

	**% Mediated**		**% Mediated**		**% Mediated**		**% Mediated**	
Cognitive function	71.7	-	45.5	-	61.2	-	53	-
Education	-1		26.4		0.5		16.3	
Vision	27.9	-	26.2	-	37.8	-	28.5	-
Hearing	3.4	-	1.9	-	0.5	-	2.3	-
**2017**								
Total effect	0.62^{**}	[0.47,0.83]	0.87	[0.62,1.20]	0.59^{***}	[0.47,0.75]	0.69^{***}	[0.53,0.90]
Direct effect	0.94	[0.71,1.26]	1.34	[0.95,1.900]	0.95	[0.74,1.21]	1.05	[0.80,1.37]
Indirect effect	0.66^{***}	[0.57,0.77]	0.65^{***}	[0.58,0.72]	0.63^{***}	[0.54,0.73]	0.66^{***}	[0.59,0.74]
**Confounding ratio and percentage**	8 (87.6%)	-	-0.50 (303.3 %)	-	9.4 (89,4%)	-	-7.6 (113.2)	-
	**% Mediated**		**% Mediated**		**% Mediated**		**% Mediated**	
Cognitive function	74.5	-	46.6	-	65.3	-	54.2	-

Education	-1		26.5		0.5		16.3	
Vision	27.9	-	24.9	-	33.1	-	27	-
Hearing	2.8	-	2.1	-	1.1	-	2.5	-
**N**	**7399**		**15916**		**7399**		**15916**	

OR Odds ratio, the reduced models were adjusted for age, sex, and country; the full models were additionally adjusted also for the mediators. Confounding ratio gives information on the total effect size relative to the direct effect size, calculated by total effect/direct effect. Confounding percentage measures the percentage change of effect attributable to confounding net of rescaling, calculated by indirect effect/total effect. * $p < 0.05$, ** $p < 0.01$, *** $p < 0.001$, Confidence intervals (CI) between brackets

Table 2. Decomposition analysis results of the total time effect on ADL and IADL limitations into direct and indirect effects through education, cognitive and sensory functions– using Karlson-Holm-Breen (KHB) method for logistic regressions, stratified by gender

	ADL male	ADL female	IADL male	IADL female
**2004-2005**				
Total effect	Base outcome	Base outcome	Base outcome	Base outcome

Direct effect	Base outcome		Base outcome		Base outcome		Base outcome	
Indirect effect	Base outcome		Base outcome		Base outcome		Base outcome	
**2006-2007**								
Total effect	0.89	[0.70,1.13]	0.88	[0.71,1.08]	0.77*	[0.61,0.96]	0.67***	[0.56,0.79]
Direct effect	1.06	[0.83,1.35]	1.07	[0.87,1.32]	0.96	[0.77,1.21]	0.81*	[0.68,0.97]
Indirect effect	0.84**	[0.75,0.93]	0.82***	[0.74,0.90]	0.80***	[0.70,0.91]	0.82***	[0.75,0.90]
**Confounding ratio and percentage**	-2 (149.1%)	-	-1.9 (152.5%)	-	6.7 (85.2%)	-	1.9 (48.1%)	-
	**% Mediated**		**% Mediated**		**% Mediated**		**% Mediated**	
Cognitive function	47.0	-	50.4	-	43.0	-	49.9	-
Vision	42.6		36		52.6		39.5	
Hearing	4.8	-	8.2	-	2.3	-	6.8	-
Education	5.5	-	5.4	-	2.2	-	3.8	-

**2011**								
Total effect	1.11	[0.84,1.46]	0.85	[0.67,1.09]	0.679**	[0.52,0.89]	0.68***	[0.56,0.83]
Direct effect	1.42*	[1.08,1.87]	1.10	[0.86,1.41]	0.916	[0.70,1.20]	0.87	[0.71,1.06]
Indirect effect	0.78***	[0.70,0.87]	0.77***	[0.70,0.86]	0.742***	[0.65,0.85]	0.78***	[0.71,0.86]
**Confounding ratio and percentage**	0.3 (-241.8%)		8.9 (88.8%)	-	4.4 (77.3%)	-	2.7 (63.2%)	-
	**% Mediated**		**% Mediated**		**% Mediated**		**% Mediated**	
Cognitive function	55.3		56.7	-	53.6	-	57.9	-
Vision	30.7		23.9		40.2		27	
Hearing	4.1		7.6	-	2.1	-	6.4	-
Education	5.5		11.8	-	4.1	-	8.7	-
**2013**								
Total effect	0.86	[0.679,1.	0.73**	[0.591,0.	0.75*	[0.60,0.9	0.58***	[0.49,0.6

		093]		906]		4]		8]
Direct effect	1.11	[0.87,1.4	0.97	[0.77,1.2	1.02	[0.82,1.2	0.75**	[0.63,0.9
		1]		1]		8]		0]
Indirect effect	0.78***	[0.70,0.8	0.76***	[0.68,0.8	0.74***	[0.65,0.8	0.77***	[0.69,0.8
		8]		5]		4]		5]
**Confounding ratio and percentage**	-1.5 (168.4%)	-	-438.5 (100.2%)	-	-12.1 (108.2%)	-	1.9 (48.3%)	-
	**% Mediated**		**% Mediated**		**% Mediated**		**% Mediated**	
Cognitive function	50.9	-	54.2	-	48.7	-	54.6	-
Vision	35.2		31.4		45.4		35	
Hearing	2	-	0.1	-	1	-	0.1	-
Education	9.9	-	14.3	-	4.9	-	10.4	-
**2015**								
Total effect	0.84	[0.66,1.0	0.69**	[0.56,0.8	0.81	[0.65,1.0	0.64***	[0.54,0.7
		7]		6]		1]		6]
Direct effect	1.19	[0.93,1.5	1.00	[0.79,1.2	1.23	[0.98,1.5	0.91	[0.76,1.0

		4]		6]		5]		9]
Indirect effect	0.70***	[0.62,0.7	0.69***	[0.62,0.7	0.66***	[0.572,0.	0.70***	[0.63,0.7
		9]		8]		75]		8]
**Confounding ratio and percentage**	-1(199.7%)	-	8.9 (88.8%)	-	1 (197.9%)	-	4.7 (78.9%)	-
	**% Mediated**		**% Mediated**		**% Mediated**		**% Mediated**	
Cognitive function	58.9	-	58.7	-	57.5	-	59.3	-
Vision	28.1		26.6		36.4		29.7	
Hearing	2.4	-	1.7	-	1.0	-	1.4	-
Education	10.7	-	13.1	-	4.4	-	9.5	-
**2017**								
Total effect	0.79	[0.59,1.0	0.67**	[0.51,0.8	0.67**	[0.50,0.8	0.60***	[0.49,0.7
		6]		8]		9]		4]
Direct effect	1.18	[0.87,1.6	1.04	[0.77,1.4	1.09	[0.82,1.4	0.92	[0.74,1.1
		0]		0]		6]		4]
Indirect effect	0.67***	[0.59,0.7	0.64***	[0.57,0.7	0.61***	[0.53,0.7	0.66***	[0.59,0.7

		5]		3]		1]		3]
**Confounding ratio and percentage**	-1.4 (170.2%)	-	-10.7 (100.4%)	-	-4.6 (121.6%)	-	5.8 (82.9%)	-
	**% Mediated**		**% Mediated**		**% Mediated**		**% Mediated**	
Cognitive function	62.5	-	58.9	-	60.5	-	60.0	-
Vision	26.9		23.7		35.1		26.7	
Hearing	0.6	-	4.5	-	0.3	-	3.9	-
Education	10.0	-	12.9	-	4.1	-	9.4	-
**N**	**10891**		**12424**		**10891**		**12424**	

OR Odds ratio, the reduced models were adjusted for age, sex, and country; the full models were additionally adjusted also for the mediators. Confounding ratio gives information on the total effect size relative to the direct effect size, calculated by total effect/direct effect. Confounding percentage measures the percentage change of effect attributable to confounding net of rescaling, calculated by indirect effect/total effect. * $p < 0.05$, ** $p < 0.01$, *** $p < 0.001$, Confidence intervals (CI) between brackets

Table 3. Regression estimates for the reduced models, adjusted for sex, gender and country

	**ADL**		**IADL**		**Hearing**		**Vision**		**Cognition**		**Education**	
	OR	95% CI	OR	95% CI	OR	95% CI	OR	95% CI	Coeff	95% CI	Coeff	95% CI
2004/ 2005	1	[1,1]	1	[1,1]	1	[1,1]	1	[1,1]	0	[0,0]	0	[0,0]
2006/ 2007	0.867	[0.744,1.009]	0.698***	[0.613,0.795]	0.875***	[0.811,0.943]	0.614***	[0.564,0.669]	0.278***	[0.223,0.333]	0.0852***	[0.0621,0.108]
2011	0.925	[0.775,1.103]	0.673***	[0.578,0.784]	0.845***	[0.776,0.921]	0.638***	[0.580,0.702]	0.420***	[0.354,0.486]	0.223***	[0.192,0.254]
2013	0.788**	[0.673,0.923]	0.651***	[0.570,0.742]	0.967	[0.893,1.048]	0.552***	[0.506,0.602]	0.399***	[0.339,0.458]	0.282***	[0.253,0.311]
2015	0.750***	[0.637,0.882]	0.704***	[0.616,0.805]	0.917*	[0.845,0.994]	0.523***	[0.479,0.570]	0.604***	[0.543,0.665]	0.349***	[0.318,0.379]
2017	0.730**	[0.594,0.897]	0.652***	[0.551,0.772]	0.884*	[0.794,0.984]	0.510***	[0.457,0.568]	0.718***	[0.636,0.799]	0.402***	[0.362,0.442]

**Age bands**												
60-64	1	[1,1]	1	[1,1]	1	[1,1]	1	[1,1]	0	[0,0]	0	[0,0]
65-69	1.072	[0.902,1.275]	1.055	[0.904,1.233]	1.154***	[1.072,1.243]	1.080	[0.999,1.167]	-0.286***	[-0.340,-0.231]	-0.0963***	[-0.128,-0.0642]
70-74	1.301*	[1.055,1.604]	1.550***	[1.300,1.847]	1.409***	[1.289,1.540]	1.337***	[1.227,1.458]	-0.676***	[-0.741,-0.610]	-0.231***	[-0.272,-0.190]
75-79	1.832***	[1.488,2.256]	2.721***	[2.282,3.245]	1.753***	[1.583,1.941]	1.755***	[1.594,1.934]	-1.143***	[-1.219,-1.066]	-0.362***	[-0.411,-0.314]
80-84	2.597***	[2.099,3.214]	4.174***	[3.484,5.002]	2.286***	[2.034,2.569]	2.455***	[2.199,2.741]	-1.653***	[-1.741,-1.564]	-0.440***	[-0.494,-0.386]
85-89	4.428***	[3.535,5.547]	7.472***	[6.146,9.084]	3.007***	[2.599,3.480]	3.597***	[3.135,4.129]	-2.189***	[-2.299,-2.078]	-0.535***	[-0.598,-0.471]
90+	8.761***	[6.659,11.53]	17.43***	[13.34,22.76]	5.135***	[4.095,6.439]	4.745***	[3.864,5.828]	-2.622***	[-2.789,-2.456]	-0.699***	[-0.780,-0.617]
**Sex**												

Male	1	[1,1]	1	[1,1]	1	[1,1]	1	[1,1]	0	[0,0]	0	[0,0]
Female	1.049	[0.919,1.199]	1.641***	[1.467,1.834]	0.558***	[0.516,0.603]	1.079*	[1.008,1.155]	0.360***	[0.300,0.419]	-0.0532*	[-0.0946,-0.0119]
**Country**												
Denmark	1	[1,1]	1	[1,1]	1	[1,1]	1	[1,1]	0	[0,0]	0	[0,0]
Sweden	0.943	[0.825,1.078]	0.786***	[0.702,0.880]	1.169***	[1.081,1.265]	1.451***	[1.356,1.553]	-0.0744*	[-0.134,-0.0148]	-0.351***	[-0.392,-0.311]
**Constant**									10.14***	[10.06,10.21]	2.133***	[2.089,2.177]
cut1					0.257***	[0.231,0.286]	0.524***	[0.474,0.579]				
cut2					0.971	[0.875,1.077]	1.882***	[1.702,2.080]				
cut3					5.594***	[4.998,6.262]	8.149***	[7.314,9.0				

								80]				
cut4					50.28***	[43.41,58.23]	28.50***	[25.09,32.37]				
**N**	**23315**		**23315**		**23315**		**23315**		**23315**		**23315**	

Exponentiated coefficients; 95% confidence intervals in brackets

* $p < 0.05$, ** $p < 0.01$, *** $p < 0.001$

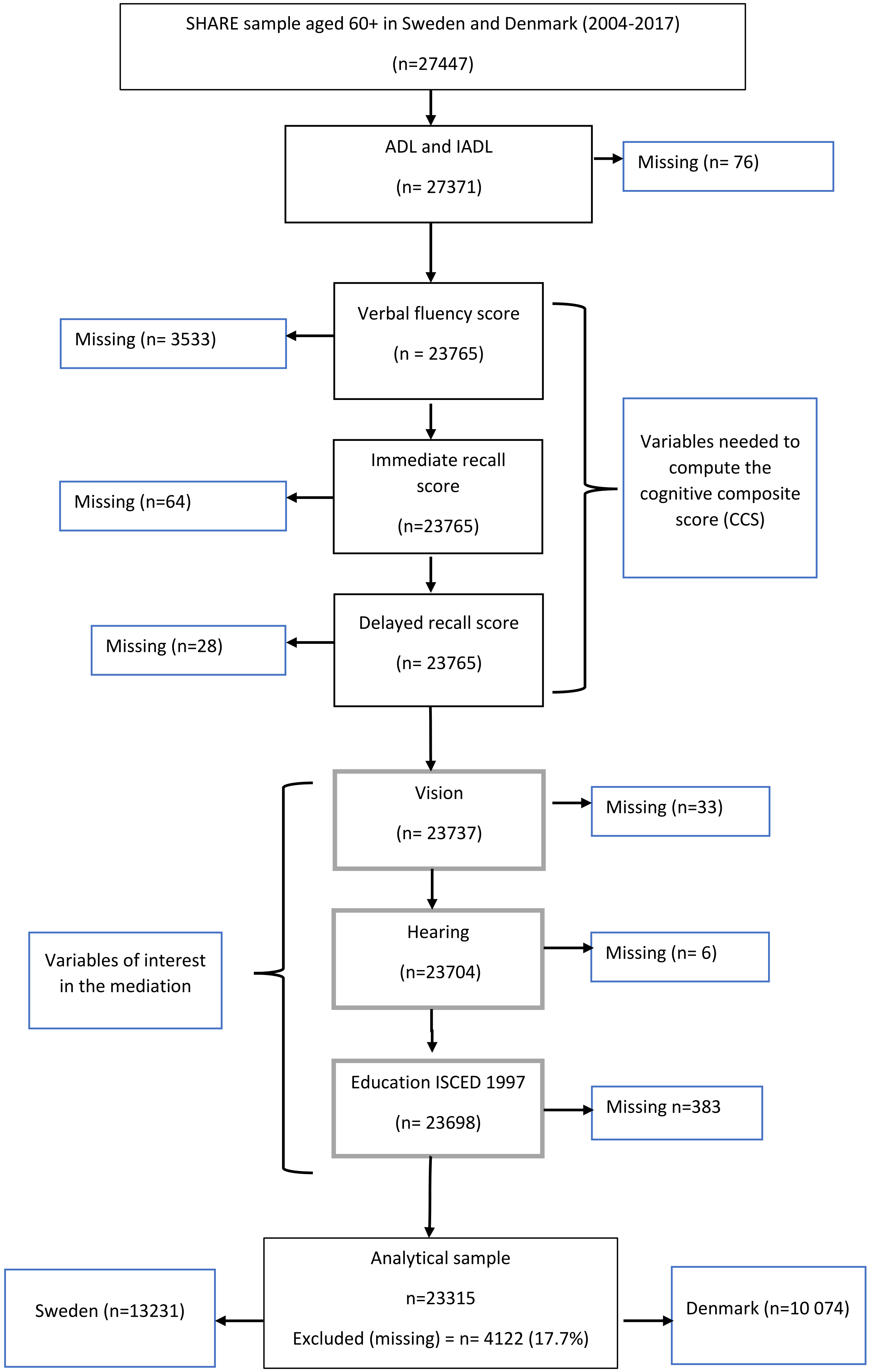

Figure 1. Flowchart the selection of the analytical sample
